# MR-Guided High-Power Microwave Ablation in Hepatic Malignancies: Initial Results in Clinical Routine

**DOI:** 10.1007/s00270-020-02584-5

**Published:** 2020-07-22

**Authors:** Moritz T. Winkelmann, Georg Gohla, Jens Kübler, Jakob Weiß, Stephan Clasen, Konstantin Nikolaou, Rüdiger Hoffmann

**Affiliations:** Department of Diagnostic and Interventional Radiology, Eberhard-Karls-University, Hoppe-Seyler-Strasse 3, 72076 Tübingen, Germany

**Keywords:** Interventional magnetic resonance imaging, Microwave ablation, Ablation techniques, Liver neoplasms, Duration of therapy

## Abstract

**Purpose:**

Evaluation of technique effectiveness, patient safety and ablation parameters of MR-guided microwave ablation in hepatic malignancies using an MR-conditional high-power microwave ablation system.

**Materials and Methods:**

Institutional review board approval and informed patient consent were obtained. Patients who underwent MR-guided microwave ablation of hepatic malignancies in a 1.5T wide-bore scanner using a perfusion-cooled high-power microwave ablation system with a maximum generator power of 150 W were included. Ablation parameters comprising procedure durations, net ablation duration, applicator positions and ablation zone dimensions were recorded. Adverse events were classified according to the CIRSE classification system. Technique effectiveness was assessed after 1 month. Follow-up was conducted with contrast-enhanced MRI and ranged from 1 to 20 months (mean: 6.1 ± 5.4 months).

**Results:**

Twenty-one consecutive patients (age: 63.4 ± 10.5 years; 5 female) underwent 22 procedures for 28 tumours (9 hepatocellular carcinomas, 19 metastases) with a mean tumour diameter of 14.6 ± 5.4 mm (range: 6–24 mm). Technique effectiveness was achieved in all lesions. Tumours were treated using 1.7 ± 0.7 applicator positions (range: 1–3). Mean energy and ablation duration per tumour were 75.3 ± 35.4 kJ and 13.3 ± 6.2 min, respectively. Coagulation zone short- and long-axis diameters were 29.1 ± 6.4 mm and 39.9 ± 7.4 mm, respectively. Average procedure duration was 146.4 ± 26.2 min (range: 98–187 min). One minor complication was reported. Five patients developed new tumour manifestations in the untreated liver. Local tumour progression was not observed during initial follow-up.

**Conclusion:**

MR-guided high-power microwave ablation provides safe and effective treatment of hepatic malignancies with short ablation times and within acceptable procedure durations.

## Introduction

Percutaneous tumour ablation is a minimally invasive treatment option for patients with hepatic malignancies who are not suitable for surgical resection due to limited hepatic function, comorbidities or unfavourable anatomic conditions [1, 2]. The most common representative of ablation techniques is radiofrequency (RF) ablation; however, several studies suggest that tumour size and proximity to large vessels are risk factors for local tumour recurrence [3–5]. In this respect, microwave ablation is an ablation technique of growing importance, which provides several physical advantages. In contrast to RF ablation, microwave ablation is independent of increasing tissue impedance during ablation and therefore enables higher intratumoral temperatures, resulting in larger ablation zones in a shorter time with a single probe [6–8]. Ex vivo studies have shown that microwave ablation is less susceptible to the heat sink effect of larger vessels, as microwave energy is directly transmitted to a defined volume [9].

Besides a reliable and effective ablation technique, precise applicator positioning and therapy monitoring are essential for successful percutaneous thermoablation. Magnetic resonance imaging (MRI) provides several advantages as a guidance modality, such as assessment of the coagulation zone without administration of contrast agent, free angulation of imaging planes, depiction of smallest hepatic lesions, MR thermometry and radiation-free near-real-time fluoroscopic sequences [10–14]. Nevertheless, restricted availability of MR scanners suitable for interventions and relatively long durations of MR-guided procedures combined with higher operating costs are the major reasons for the limited acceptance of MR-guided ablation procedures in clinical routine [15]. Previous MR-guided microwave ablations of hepatic malignancies have been performed at our institution using a low-power ablation system with high technical efficiency but relatively long procedure durations [16]. In this context, a high-power ablation system capable of creating large ablation zones in a short time is of particular interest, especially in combination with MR guidance.

The aim of this study was the evaluation of technique effectiveness, patient safety and ablation parameters of MR-guided microwave ablation in hepatic malignancies using an MR-conditional high-power microwave ablation system.

## Materials and Methods

### Patients

Institutional review board approval and informed patient consent were obtained for this prospective single-centre study. Patients who underwent MR-guided microwave ablation of a primary or secondary hepatic malignancy between June 2018 and December 2019 were included. Hepatic tumour ablations are routinely conducted under MR guidance at our institution, so that ablation procedures in the liver are only conducted under CT guidance, if contraindications to MRI exist. All procedures were conducted after decision in an interdisciplinary tumour board. Internal guidelines for percutaneous tumour ablation include a maximum of three liver metastases or hepatocellular carcinoma (HCC) lesions and a maximum tumour diameter of 5 cm. Further preconditions for percutaneous tumour ablation are appropriate location of the target tumour (e.g. sufficient distance from the liver hilum or heat-sensitive organs) and adequate laboratory results (INR < 1.5; platelet count ≥ 50,000/µL).

### Technical Equipment

All ablations were conducted with a high-power microwave ablation system with a maximum generator power of 150 W and a generator frequency of 2.45 GHz. The system is equipped with a perfusion pump for cooling of the applicator shaft. All ablations were performed with an MR-compatible, 14-G microwave applicator (ECO-100AI13C, Nanjing ECO Medical Instrument Co., China) with a length of 15 cm. The applicator is composed of a shaft based on titanium alloy and a ceramic tip. The microwave generator (ECO-100E2, Nanjing ECO Medical Instrument Co, China) was positioned outside the scanner room during the procedures, and a 4-m-long coaxial cable connected the generator with the MR-compatible antenna.

The procedures were conducted in one of two wide-bore 1.5-T systems (Siemens MAGNETOM Espree and Siemens MAGNETOM Aera, Siemens Healthineers, Erlangen, Germany) equipped for MR-guided interventions. An RF-shielded liquid crystal display monitor was positioned next to the scanner’s bore and enabled real-time monitoring during the intervention while the interventionalist was sitting close to the patient lying in the scanner.

### Patient Treatment

**Fig. 1:**
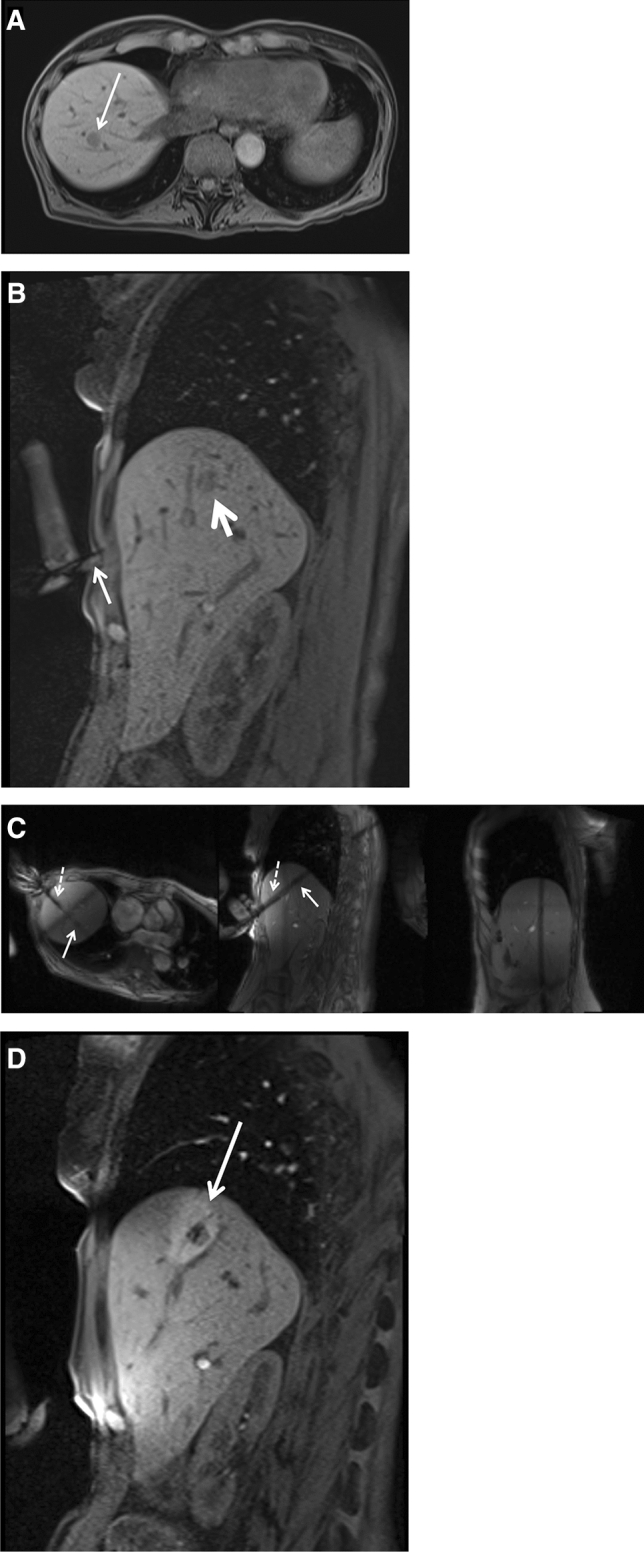
Sixty-one-year-old female patient with hepatic metastases from a neuroendocrine tumour. Planning imaging with unenhanced T1-weighted VIBE **A** reveals a 12-mm metastasis in segment VIII (arrow). Coronal T1-weighted VIBE **B** depicts the small tip artefact of the microwave applicator in the subcutaneous tissue (small arrow) pointing towards the target lesion (larger arrow). Near-real-time imaging with MR fluoroscopy **C** shows the microwave antenna (dotted arrows) in three slice angulations and enables an accurate targeting of the tumour (arrows). Coronal therapy monitoring with T1-weighted VIBE **D** shows a hyperintense ablation zone covering the target tumour with an adequate safety margin

The whole procedure including planning imaging, tumour targeting, therapy monitoring and control imaging were conducted with the patient positioned on the scanner table. Procedures were conducted under analgesia in 8 cases (8–15 mg piritramide i.v) or under general anaesthesia in 14 cases. The potential cutaneous puncture point was marked with a capsule (Nifedipine AL 5, Aliud Pharma, Laichingen, Germany) and unenhanced planning sequences were acquired (Fig. 1A). Detailed interventional sequence information is displayed in Table 1. Additional diffusion-weighted imaging (DWI) or contrast-enhanced 3D T1-weighted Dixon volumetric interpolated breath-hold examinations VIBE were acquired after intravenous injection of 0.025 mmol/kg body weight of gadoxetate disodium (Primovist, Bayer HealthCare, Germany), if the target tumour visualization was insufficient during standard planning imaging. After disinfection, a six-channel body array coil was placed at the puncture site, so that the applicator was positioned through one of four holes in the body array coil. In case of a lateral access, the puncture was conducted through an additional loop coil. After sterile draping and subcutaneous local anaesthesia (Xylocaine 1%, AstraZeneca, Wedel, Germany), a small skin incision was made at the entry point. 3D T1-weighted VIBE was acquired with the microwave applicator placed in the subcutaneous tissue (Fig. 1B). This sequence was used to adjust the imaging slices of a MR fluoroscopic sequence (BEAT-Multislice), which allows near-real-time tracking of the applicator in three imaging orientations during tumour targeting (Fig. 1C). 3D T1-weighted VIBE was repeated to confirm the correct applicator position. The previously reported impaired visibility of the applicator tip had to be considered during applicator placement and position control [17]. Ablation was conducted after connection of the microwave antenna and the generator via an MR-compatible coaxial cable with the generator placed outside the scanner room. Ablation settings were selected according to the results of initial ex vivo experiments in bovine liver with the generator power ranging from 80 to 100 W [17]. After ablation, unenhanced 3D T1-weighted VIBE was acquired for therapy monitoring without having withdrawn the microwave antenna (Fig. 1D). If the ablation zone, which depicts as a hyperintense area in T1-weighted imaging, was considered insufficient, ablation was repeated with the same applicator position or after repositioning of the applicator. If the ablation zone was considered adequate with full coverage of the target tumour including a sufficient safety margin of > 5 mm, the applicator was retracted under coagulation. Post-interventional control imaging including axial T2-weighted TSE sequence and multiphasic contrast-enhanced 3D T1-weighted Dixon VIBE after intravenous injection of gadobutrol 0.1 mmol/kg body weight (Gadovist, Bayer HealthCare) was performed to evaluate technical success and to exclude complications.

**Table 1:** Sequence details

Sequence	TE (ms)	TR (ms)	Slice thickness (mm)	Matrix (mm^2^)	Flip angle (°)	Bandwidth (Hz/pixel)
*Planning*						
T2 HASTE (coronal)	94	1100	3	256 × 120	160	490
T2 TSE	95	5800	6	384 × 384	158	590
T1 Dixon VIBE	2.4	6.7	3	320 × 182	10	520
Diffusion*	59	3000	6	192 × 144	90	1860
CE dynamic T1 Dixon VIBE^a,^*	2.4	6.7	3	320 × 182	10	520
*Targeting*						
BEAT-Multislice	3.2	464	8	128 × 128	20	500
*Therapy monitoring*						
T1 VIBE	1.3	3.5	2	320 × 189	10	400
T1 Flash 2D	4.1	193	6	256 × 140	70	140
*Control*						
T2 TSE	95	5800	6	384 × 384	158	590
CE dynamic T1 Dixon VIBE^b^	2.4	6.7	3	320 × 182	10	520

*HASTE* half acquisition single shot turbo spin echo, *TSE* turbo spin echo, *VIBE* volumetric interpolated breath-hold imaging*, BEAT*-*Multislice* multiplanar interactive real-time sequence, *Flash* fast low angle shot gradient echo, *TrueFISP* True fast imaging with steady state free precession, *Diffusion* echoplanar imaging with *b*-values of 0, 400, 800 mm/s^2^, *CE* contrast enhanced

^a^After i.v. injection of 0.025 mmol/kg body weight of gadoxetate disodium

^b^After i.v. injection of 0.1 mmol/kg body weight of gadobutrol

*Optional

### Follow-Up Imaging

Internal Institutional guidelines recommend abdominal ultrasound 1 day post-ablation to exclude bleeding or cholestasis. The further follow-up scheme includes contrast-enhanced liver MRI 1 month after ablation and every 3 months for 1 year. Contrast-enhanced liver MRI is repeated every 6 months thereafter. The following sequences were acquired: coronal T2-weighted half acquisition single shot turbo spin echo (HASTE), axial T2-weighted TSE with navigator technique, echo planar imaging (EPI) for diffusion-weighted imaging with *b*-values of 0, 400 and 800 s/mm^2^ and T1-weighted Dixon VIBE dynamic liver examination after intravenous injection of gadobutrol (Gadovist) 0.1 mmol/kg body weight.

### Data Analysis

Technical success was assessed based on contrast-enhanced control imaging at the end of the intervention. The procedure was considered technically successful if the non-enhancing ablation zone covered the target tumour without evidence of residual tumour [18]. The short axis diameter (SAD) and long axis diameter (LAD) of the ablation zone were measured on the contrast-enhanced 3D T1-weighted VIBE sequence at the end of the intervention using post-processing software (syngo.via, Siemens Healthineers). The sphericity index (SI) was calculated as SAD/LAD. Technique effectiveness was defined as complete ablation at initial follow-up imaging 1 month after treatment. Suspicion of tumour adjacent to the ablation zone after 4 months and later was considered as local tumour progression [19]. Adverse events were based on electronic patient records and classified according to the CIRSE classification of complications [20]. Ablation parameters were collected from a standardized report completed by the interventionist after treatment. Procedure duration was defined as time between acquisition of the initial localizer sequence and the final control sequence.

## Results

### Patient Characteristics and Ablation Parameters

Between June 2018 and November 2019, 21 consecutive patients (5 female, 16 male) were prospectively included in this study and underwent MR-guided microwave ablation of hepatic malignancies. Mean patient age was 63.4 ± 10.5 years (range: 40–79 years). A total of 28 lesions were treated in 22 interventions, including 17 interventions on a single lesion and 5 interventions on two lesions. The mean lesion size was 14.6 ± 5.4 mm (range: 6–24 mm). Table 2 states further patient and tumour characteristics.

**Table 2:** Patient und tumour characteristics

Patient no./age (years)	Tumour no.	Liver segment	Tumour entity	Maximal tumour diameter (mm)
1/47	1.1^a^	V	Metastasis uveal melanoma	17
	1.2^a^	VIII	Metastasis uveal melanoma	13
2/66	2	VIII^c^	HCC^b^	20
3/66	3	VIII	HCC^b^	15
4/64	4	IV	Metastasis CRC	21
5/57	5	VIII^c^	Metastasis CRC	12
6/72	6.1	VIII	HCC^b^	15
	6.2	V	HCC^b^	12
7/48	7	VII^c^	HCC^b^	25
8/54	8	VIII^c^	HCC^b^	21
9/65	9	VI	Metastasis uveal melanoma	10
10/62	10	VIII	Metastasis CRC	8
11/61	11.1	VIII	Metastasis NET	12
	11.2	VIII	Metastasis NET	6
12/64	12.1	VIII^c^	Metastasis CRC	21
	12.2	V	Metastasis CRC	6
13/61	13.1	IV	Metastasis CRC	7
	13.2	VII	Metastasis CRC	8
14/72	14	VIII^c^	HCC	14
15/40	15	VIII	Metastasis pancreas carcinoma	24
16/77	16	VIII	Metastasis uveal melanoma	13
17/72	17.1	IV^c^	HCC^b^	17
	17.2	VIII	HCC^b^	15
18/79	18	V	Metastasis CRC	24
19/74	19	VI	Metastasis CRC	12
20/60	20.1	II^c^	Metastasis CRC	16
	20.2	VII	Metastasis CRC	14
21/77	21	V	Metastasis CRC	10

*HCC* hepatocellular carcinoma, *CRC* colorectal carcinoma, *NET* neuroendocrine tumour

^a^tumours treated in two procedures, ^b^underlying cirrhosis, ^c^subcapsular tumour location

The lesions were treated using 1.7 ± 0.7 applicator positions (range 1–3). Thirteen target tumours were treated with a single applicator position (46%). A mean energy of 75.3 ± 35.4 kJ was applied per tumour (range: 18–150 kJ) within a mean application duration of 13.3 ± 6.2 min (range 3–26 min). Mean short axis and long axis diameter of the ablation zone were 29.1 ± 6.4 mm (range: 19–40 mm) and 39.9 ± 7.4 mm (range: 28–58 mm), respectively, resulting in a mean sphericity index of 0.74. The mean sphericity index of the ablation zones without applicator repositioning (*n* = 13) measured 0.7. Average duration of the interventions, including planning and control imaging, was 146.4 ± 26.2 min (range: 98–187 min). Average duration of interventions with one target lesion (n = 17) was 137.7 ± 25.2 min. Intra-procedural planning imaging was extended by DWI in three procedures (14%). Additional contrast-enhanced dynamic imaging after administration of gadoxetate disodium was required in four procedures (18%) to improve visibility of the target lesion.

### Technical Success, Technique Effectiveness and Local Tumour Progression

28/28 tumours were completely ablated, corresponding to a technical success rate of 100%. None of the ablation zones showed signs of residual tumour in follow-up imaging after 1 month, resulting in a primary technique effectiveness of 100%. Duration of follow-up ranged from 1 to 20 months (mean: 6.1 ± 5.4 months). During initial follow-up, no case of local tumour progression was detected. However, five patients developed new tumour manifestations in the untreated liver, three of whom were subsequently treated with chemotherapy. One of these patients with colorectal liver metastases was initially planned for a second microwave ablation of one new hepatic metastasis. However, in intra-procedural planning imaging with DWI several small new metastases were detected in this patient, so that the procedure was not performed and systemic therapy was initiated. One patient with uveal melanoma underwent a second microwave ablation of a single new hepatic lesion, but developed further hepatic metastases during follow-up and was treated with hepatic chemosaturation. Another patient with uveal melanoma and two new hepatic metastases is planned for a second microwave ablation at the time of manuscript preparation.

### Complications and Side Effects

One patient developed a rash on the face and trunk immediately after control imaging as a side-effect of contrast agent administration. The symptoms subsided after intravenous administration of antihistamines and prednisolone. This event was classified as a grade 1 minor complication according to the CIRSE classification. No further complications occurred. All patients were discharged after a maximum of 2 days hospitalization (mean: 1.5 ± 0.5 days).

## Discussion

In our study, total procedure durations were shorter than in comparable studies concerning MR-guided tumour ablation. Rempp et al. reported average procedure durations of 3.7 h using MR-guided RF ablation for the treatment of hepatic malignancies with a mean diameter of 20 mm [21]. Although several studies have shown distinctly shorter ablation times with microwave ablation compared to RF ablation, an obvious time-saving effect could not be confirmed in our first study concerning MR-guided liver ablation from 2017 with mean procedure durations above 3 h per tumour [16, 22, 23]. In this older study, 18 hepatic tumours with a mean diameter of 15 mm were treated using a non-perfusion cooled, low-power ablation system with a maximum power of 36 W. In comparison with this study, we report shorter procedure durations of 137 min for the treatment of a single tumour which may be explained by two factors. In the historical study, 2.5 applicator positions on average were necessary to treat one tumour and net ablation duration per tumour was 24.7 min. In comparison, almost half of target tumours could be treated without repositioning of the applicator in our study and net ablation time per tumour was 13.3 min. These differences are well explained by a porcine in vivo study by Bedoya et al., which showed that microwave ablation with a higher output power creates larger ablation zones with larger short axis diameters in a shorter time despite the same energy amounts [24]. In this context, several publications claim higher local effectivity of high-power microwave ablation systems as relatively more heat will be dispersed into the surrounding tissue [25, 26]. In our preliminary study, local recurrence was not observed which may underline this theory. However, the follow-up period and patient numbers are too low for such conclusions.

In our study, DWI or gadoxetic acid-enhanced sequences were additionally acquired in seven cases for planning imaging. These additional sequences are relatively time-consuming, especially since the time until a sufficient hepatobiliary phase for detection of small tumours is reached can significantly affect the procedure duration, as it depends on the hepatic function [27]. Nevertheless, these additional sequences can be relevant for detection and targeting of small tumours and can affect therapy decisions, as shown in our study where several new small metastases were detected in one patient using DWI during planning imaging. Fischbach et al. optimized procedure durations for MR-guided thermoablation and routinely administered hepatocyte-specific contrast agent 20 min before the procedure [28]. However, in our series most target tumours were visible without the use of contrast agent, and pre-interventional administration of gadoxetic acid would not have been necessary for treatment. Consequently, we decided to consider pre-interventional administration of gadoxetic acid in further procedures in cases where a benefit is expected from pre-interventional imaging.

No major complications occurred in our study, and only one minor complication related to intravenous contrast administration was reported. Nevertheless, one relevant issue regarding patient safety concerns the visualization of the microwave applicator. In our recently reported ex vivo evaluation of the appearance of the applicator’s MR artefact, the applicator shaft was clearly visible under MR guidance while the tip only showed a small artefact [17]. This observation was even stronger under clinical, non-optimal conditions, especially with MR-fluoroscopic sequences. Future augmentation of the tip artefact would ease applicator positioning, increase patient safety and may finally shorten procedure durations as intermittent control scans could be avoided.

The following limitations of our study need to be addressed. First, the patient cohort of this preliminary study is relatively small and the follow-up period is too short for a reliable assessment of local tumour control. No histological sampling was performed prior to ablation to confirm the diagnosis. Furthermore, the treated tumours were relatively small. Consequently, further studies with a longer follow-up period and larger tumours are necessary.

## Conclusion

MR-guided perfusion-cooled high-power microwave ablation is safe and enables a high primary effectiveness regarding the ablation of smaller tumours and can be conducted within acceptable procedure durations. However, future studies are necessary to evaluate the potential of this method, especially regarding the treatment of larger tumours.

## References

[1] Gillams A, Goldberg N, Ahmed M, Bale R, Breen D, Callstrom M (2015). Thermal ablation of colorectal liver metastases: a position paper by an international panel of ablation experts, the Interventional Oncology Sans Frontiers meeting 2013. Eur Radiol.

[2] Meijerink MR, Puijk RS, van Tilborg A, Henningsen KH, Fernandez LG, Neyt M (2018). Radiofrequency and microwave ablation compared to systemic chemotherapy and to partial hepatectomy in the treatment of colorectal liver metastases: a systematic review and meta-analysis. Cardiovasc Interv Radiol.

[3] Frericks BB, Ritz JP, Albrecht T, Valdeig S, Schenk A, Wolf KJ (2008). Influence of intrahepatic vessels on volume and shape of percutaneous thermal ablation zones: in vivo evaluation in a porcine model. Investig Radiol.

[4] Lee HY, Rhim H, Lee MW, Kim YS, Choi D, Park MJ (2013). Early diffuse recurrence of hepatocellular carcinoma after percutaneous radiofrequency ablation: analysis of risk factors. Eur Radiol.

[5] Cortis K, Degiorgio S, Cannataci C, Miraglia R (2017). Balloon-occluded microwave ablation: a potential therapeutic option in liver lesions bearing close proximity to major hepatic and/or portal veins. Cardiovasc Interv Radiol.

[6] Fan W, Li X, Zhang L, Jiang H, Zhang J (2012). Comparison of microwave ablation and multipolar radiofrequency ablation in vivo using two internally cooled probes. Am J Roentgenol.

[7] Laeseke PF, Lee FT, Sampson LA, van der Weide DW, Brace CL (2009). Microwave ablation versus radiofrequency ablation in the kidney: high-power triaxial antennas create larger ablation zones than similarly sized internally cooled electrodes. J Vasc Interv Radiol.

[8] Di Vece F, Tombesi P, Ermili F, Maraldi C, Sartori S (2014). Coagulation areas produced by cool-tip radiofrequency ablation and microwave ablation using a device to decrease back-heating effects: a prospective pilot study. Cardiovasc Interv Radiol.

[9] Primavesi F, Swierczynski S, Klieser E, Kiesslich T, Jager T, Urbas R (2018). Thermographic real-time-monitoring of surgical radiofrequency and microwave ablation in a perfused porcine liver model. Oncol Lett.

[10] Ricke J, Thormann M, Ludewig M, Jungnickel K, Grosser O, Wybranski C (2010). MR-guided liver tumor ablation employing open high-field 1.0T MRI for image-guided brachytherapy. Eur Radiol.

[11] Weiss J, Hoffmann R, Rempp H, Kebetaler DE, Pereira PL, Nikolaou K (2019). Feasibility, efficacy, and safety of percutaneous MR-guided ablation of small (</=12 mm) hepatic malignancies. J Magn Reson Imaging.

[12] Pediconi F, Marzocca F, Cavallo Marincola B, Napoli A (2018). MRI-guided treatment in the breast. J Magn Reson Imaging.

[13] Kuroda K (2018). MR techniques for guiding high-intensity focused ultrasound (HIFU) treatments. J Magn Reson Imaging.

[14] Streitparth F, Walter T, Wonneberger U, Schnackenburg B, Philipp CM, Collettini F (2014). MR guidance and thermometry of percutaneous laser disc decompression in open MRI: an ex vivo study. Cardiovasc Interv Radiol.

[15] Maurer MH, Schreiter N, de Bucourt M, Grieser C, Renz DM, Hartwig T (2013). Cost comparison of nerve root infiltration of the lumbar spine under MRI and CT guidance. Eur Radiol.

[16] Hoffmann R, Rempp H, Kessler DE, Weiss J, Pereira PL, Nikolaou K (2017). MR-guided microwave ablation in hepatic tumours: initial results in clinical routine. Eur Radiol.

[17] Grimm A, Winkelmann M, Weiss J, Gohla G, Blumenstock G, Nikolaou K (2019). Artefact and ablation performance of an MR-conditional high-power microwave system in bovine livers: an ex vivo study. Eur Radiol Exp.

[18] Kierans AS, Elazzazi M, Braga L, Leonardou P, Gerber DA, Burke C (2010). Thermoablative treatments for malignant liver lesions: 10-year experience of MRI appearances of treatment response. Am J Roentgenol.

[19] Ahmed M, Solbiati L, Brace CL, Breen DJ, Callstrom MR, Charboneau JW (2014). Image-guided tumor ablation: standardization of terminology and reporting criteria—a 10-year update. Radiology.

[20] Filippiadis DK, Binkert C, Pellerin O, Hoffmann RT, Krajina A, Pereira PL (2017). Cirse quality assurance document and standards for classification of complications: the Cirse classification system. Cardiovasc Interv Radiol.

[21] Rempp H, Waibel L, Hoffmann R, Claussen CD, Pereira PL, Clasen S (2012). MR-guided radiofrequency ablation using a wide-bore 1.5-T MR system: clinical results of 213 treated liver lesions. Eur Radiol.

[22] Takahashi H, Kahramangil B, Kose E, Berber E (2018). A comparison of microwave thermosphere versus radiofrequency thermal ablation in the treatment of colorectal liver metastases. HPB (Oxford).

[23] Sparchez Z, Mocan T, Hajjar NA, Bartos A, Hagiu C, Matei D (2019). Percutaneous ultrasound guided radiofrequency and microwave ablation in the treatment of hepatic metastases. A monocentric initial experience. Med Ultrason.

[24] Bedoya M, del Rio AM, Chiang J, Brace CL (2014). Microwave ablation energy delivery: influence of power pulsing on ablation results in an ex vivo and in vivo liver model. Med Phys.

[25] Ruiter SJS, Heerink WJ, de Jong KP (2019). Liver microwave ablation: a systematic review of various FDA-approved systems. Eur Radiol.

[26] Meloni MF, Chiang J, Laeseke PF, Dietrich CF, Sannino A, Solbiati M (2017). Microwave ablation in primary and secondary liver tumours: technical and clinical approaches. Int J Hyperthermia.

[27] Kim JE, Kim HO, Bae K, Choi DS, Nickel D (2019). T1 mapping for liver function evaluation in gadoxetic acid-enhanced MR imaging: comparison of look-locker inversion recovery and B1 inhomogeneity-corrected variable flip angle method. Eur Radiol.

[28] Fischbach F, Lohfink K, Gaffke G, Wybranski C, Mohnike K, Wonneberger U (2013). Magnetic resonance-guided freehand radiofrequency ablation of malignant liver lesions: a new simplified and time-efficient approach using an interactive open magnetic resonance scan platform and hepatocyte-specific contrast agent. Investig Radiol.

